# Evolution of CCR5 and CCR2 Genes in Bats Showed Multiple Independent Gene Conversion Events

**DOI:** 10.3390/v14020169

**Published:** 2022-01-18

**Authors:** Alexandre P. Fernandes, Ana Águeda-Pinto, Ana Pinheiro, Hugo Rebelo, Pedro J. Esteves

**Affiliations:** 1 CIBIO, Centro de Investigação em Biodiversidade e Recursos Genéticos, InBIO Laboratório Associado, Campus de Vairão, Universidade do Porto, 4485-661 Vairao, Portugal; up201900698@edu.fc.up.pt (A.P.F.); anaagueda@cibio.up.pt (A.Á.-P.); ana.pinheiro@cibio.up.pt (A.P.); hugo.rebelo@cibio.up.pt (H.R.); 2 Departamento de Biologia, Faculdade de Ciências, Universidade do Porto, 4099-002 Porto, Portugal; 3 BIOPOLIS Program in Genomics, Biodiversity and Land Planning, CIBIO, Campus de Vairão, 4485-661 Vairao, Portugal; 4 CIBIO/InBIO, Universidade de Lisboa, Tapada da Ajuda, 1349-017 Lisboa, Portugal; 5 CITS-Centro de Investigação em Tecnologias da Saúde, Instituto Politécnico de Saúde do Norte (IPSN), Cooperativa de Ensino Superior Politécnico e Universitário (CESPU), 4585-116 Gandra, Portugal

**Keywords:** chiroptera order, CCR proteins, chemokine receptors, gene conversion

## Abstract

Chemokine receptors are an important determinant for the infectiousness of different pathogens, which are able to target the host cells by binding to the extracellular domains of these proteins. This is the mechanism of infection of HIV-1, among other concerning human diseases. Over the past years, it has been shown that two chemokine receptors, CCR2 and CCR5, have been shaped by events of gene conversion in different mammalian lineages, which has been linked to a possible selective advantage against pathogens. Here, by taking advantage of available bat genomes, we present the first insight of CCR2 and CCR5 evolution within the Chiroptera order. In total, four independent events of recombination between CCR2 and CCR5 were detected: two in a single species, *Miniopterus natalensis*; one in two species from the Rhinolophoidea superfamily; and one in four species from the Pteropodidae family. The regions affected by the gene conversions were generally extensive and always encompassed extracellular domains. Overall, we demonstrate that CCR2 and CCR5 have been subject to extensive gene conversion in multiple species of bats. Considering that bats are known to be large reservoirs of virus in nature, these results might indicate that chimeric *CCR2*-*CCR5* genes might grant some bat species a selective advantage against viruses that rely in the extracellular portions of either CCR2 or CCR5 as gateways into the cell.

## 1. Introduction

Chemokine receptors are a family of seven-transmembrane-spanning G proteins important to immunological pathway signaling due to their binding ability with chemokines. They are composed of an extracellular N-terminus, an intracellular C-terminus and seven transmembrane helices (TM1-7), which are intercalated by three intracellular (ICL1-3) and three extracellular (ECL1-3) loops [1]. Many of these proteins are known to form homodimers or heterodimers, enabling new signaling pathways or enhancing already existing ones [2,3]. Chemokine receptors are expressed in lymphocytes, neutrophils, dendritic cells, and many other cell types, and play a part in both the innate and adaptive immune responses, being mainly involved in the trafficking of leukocyte populations to a site of injury and/or infection [1]. Additionally, some of these receptors have been deemed as determinants of infectiousness for different pathogens [4,5,6,7,8,9,10,11]. Most notably, CCR5 and CXCR4 have both been identified as vital co-receptors that mediate human immunodeficiency virus (HIV) entry into human cells [10,11,12,13].

Over the past years, it was shown that gene conversion events have shaped two chemokine receptors, CCR2 and CCR5, in several clades of mammals [14,15,16,17,18,19]. These genes are particularly prone to events of recombination due to their high degree of similarity (~76% identity for the human CCR2 and CCR5) and because of their proximity in the genome [1]. Two hypotheses have been put forward as possible explanations for the fixation of chimeric CCR2-CCR5 genes in different orders of mammals: (i) they facilitate the formation of CCR2-CCR5 heterodimers, which have been shown to naturally occur in human cells [14]; or (ii) they provide a selective advantage against viruses which may have used either CCR5 or CCR2 as gateways to enter the cell (in a similar fashion to HIV-1 in humans) [15,16,17]. So far, such CCR2-CCR5 recombinants have been reported in rodent, feline, leporid, guinea pig, horse, pig, marsupial, and primate lineages [14,15,16,17,18,19]. However, no evolutionary studies regarding these genes have been performed in bat species.

Bats species (Chiroptera order) have been frequently suggested as the source of outbreaks of multiple human zoonotic diseases, making them useful animal models for the fields of immunology and virology. Some examples of such diseases include rabies, Marburg virus, Ebola virus, Middle East respiratory syndrome coronavirus, and, more recently, severe acute respiratory syndrome coronavirus-2, all of which are believed to have recent ancestry in bat species and to have later been transmitted to humans by virus spillover [20,21,22,23]. Because bat colonies represent one of the largest natural reservoirs of virus in the animal kingdom [24,25], it is of the utmost importance to understand how their immune system interacts and co-evolves with viruses. In this study, we investigate possible events of gene conversion happening in CCR2 and CCR5 genes from different bat species, which may pave the way to further clarify their immune system function on these mammals.

## 2. Materials and Methods

### 2.1. Data Retrieval

The coding DNA sequences (CDSs) of CCR2 and CCR5 genes from 18 bat species were manually extracted from the NCBI database (https://www.ncbi.nlm.nih.gov/, accessed on 22 December 2021). In one species, *Myotis lucifugus*, the CCR2 gene was found as a pseudogene and, therefore, it was not included in our dataset. For the remaining species, a single copy of both CCR2 and CCR5 was found. Additionally, *Homo sapiens, Macaca mulatta, Canis lupus familiaris* and *Choloepus didactylus* CCR2 and CCR5 genes were included in the dataset as outgroups, resulting in a final dataset of 43 sequences. The sequences were then aligned using the ClustalW2 algorithm [26] with a gap opening penalty of 10 and a gap extending penalty of 0.2, followed by manual corrections where necessary.

### 2.2. Detection of Recombination and Phylogenetic Analysis

The obtained nucleotide alignment was used to create a Maximum Likelihood (ML) phylogenetic tree with the MEGA software [27,28] and 1000 bootstrap repetitions. The Tamura 3-parameter model with five gamma categories was selected as the best model of substitution for the tree by the BIC (Bayesian Information Criterion) implemented in MEGA.

For the detection of recombination events, the sequence alignment dataset was analyzed under six different algorithms (3Seq, SiScan, Chimaera, MaxChi, GENECONV and RPD) implemented in the Recombination Detection Program (RDP) [29]. To keep a conservative approach, only events of recombination detected by at least five of the six algorithms implemented were considered significant (Table S1).

## 3. Results and Discussion

**Figure 1:**
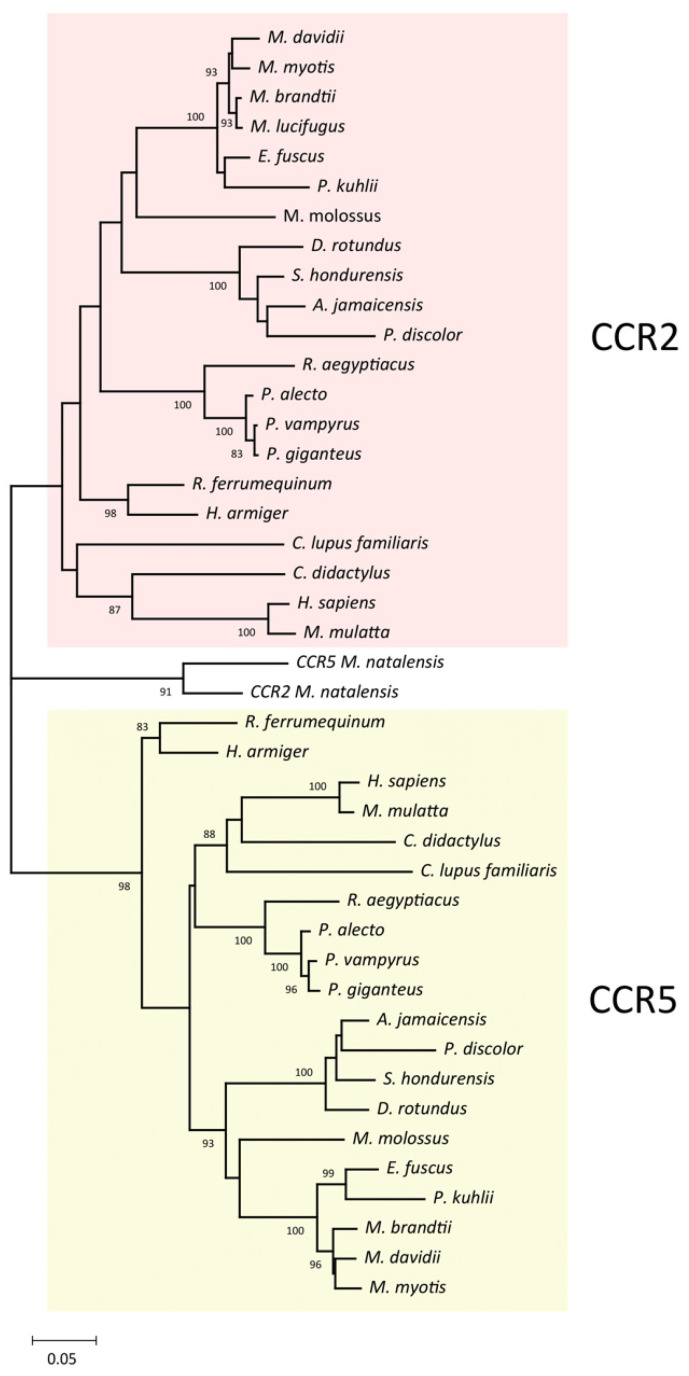
Phylogenetic analysis of CCR5 and CCR2 nucleotide sequences from different bat species. The Maximum Likelihood tree of bat CCR2 and CCR5 nucleotide sequences was generated with MEGA [27,28] using 1000 bootstrap repetitions. Only bootstrap values >75% are shown.

In this study, our main goal was to determine whether the CCR2 and CCR5 genes of bats were affected by gene conversion during their evolutionary history. For that, CCR2 and CCR5 CDSs from 18 different bat species were retrieved from public databases (Figure 1). Bats included in this study represent a wide variety of species, coming from seven different major Chiroptera families: Rhinolophidae (one species), Hipposideridae (one species), Pteropodidae (four species), Phyllostomidae (four species), Molossidae (one species), Miniopteridae (one species) and Vespertilionidae (six species). As can be observed from Figure 1, the obtained ML tree could not solve the phylogeny of the sequences at its root, causing it to be split in three different clusters supported by high bootstraps: one containing the CCR5 and CCR2 of *Miniopterus natalensis* (91 bootstrap value), one containing the remaining *CCR5* sequences, and one containing the remaining CCR2 CDSs sequences. The fact that *M. natalensis* CCR2 and CCR5 genes were clustered together in a separate group indicate that these sequences are more similar to each other than to their respective orthologs, which prompted us to investigate whether gene conversion could explain the phylogeny of the obtained tree.

**Figure 2:**
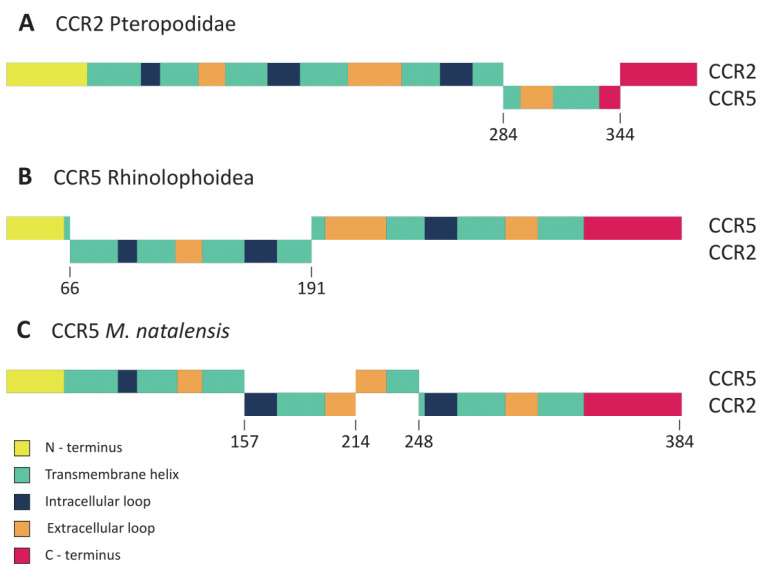
Diagrams of CCR2 and CCR5 genes detected to have undergone gene conversion in bats. RDP [29] analyses performed with the CDSs of CCR2 and CCR5 genes from Chiroptera species have identified four independent events of gene conversion to have occurred in this order. Sections of the proteins which have been acquired through gene conversion are shown in the diagrams as dislocated blocks with their starting and ending positions marked. Protein domains are colored according to the legend. (**A**) CCR2 protein of Pteropodidae family members. (**B**) CCR5 protein of the Rhinolophoidea superfamily members. (**C**) CCR5 protein of M. natalensis.

**Figure 3:**
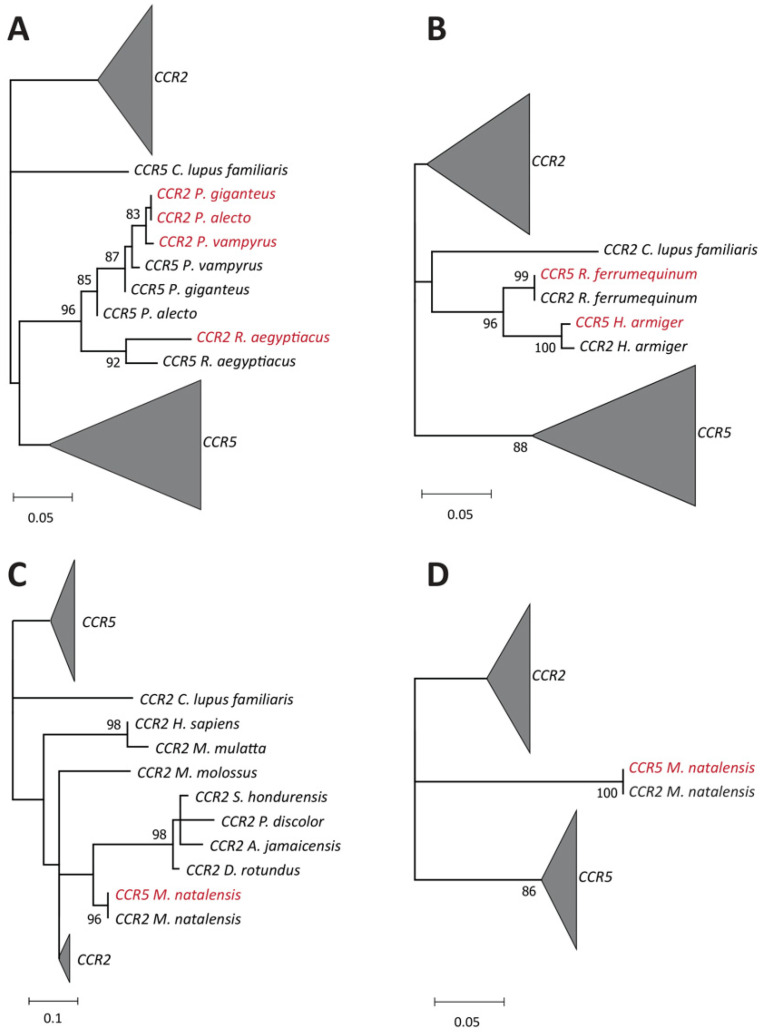
ML trees generated using only regions affected by each event of gene conversion detected in bat species. ML trees were generated with 1000 bootstrap repetitions using MEGA [27,28]. Only bootstraps values above 75% are shown below each branch. Recombinant sequences are marked in red in the ML trees. (**A**) ML tree of the subsection of the alignment where recombination has shaped the CCR2 of the Pteropodidae family (positions 284 to 344). (**B**) ML tree of the subsection of the alignment where recombination has shaped the CCR5 of the Rhinolophoidea superfamily (positions 66 to 191). (**C**) ML tree of the first subsection of the alignment where recombination has shaped the CCR5 of *M. natalensis* (positions 157 to 214). (**D**) ML tree of the second subsection of the alignment where recombination has shaped the CCR5 of *M. natalensis* (positions 248 to 384).

To search for signals of gene conversion in our dataset, we used the RDP software [29] to analyze the sequences under different algorithms (see Section 2). This method identified four different events of gene conversion that have independently occurred in ancestors of bats (Figure 2 and Table S1). Individual phylogenetic trees were then created for each event of gene conversion using only the regions of the alignment identified to be involved in recombination (Figure 3). These trees were used along with statistics provided by RDP to infer the direction of gene conversion (whether CCR5 was converted to CCR2 or the opposite) for each of the recombination events.

**Figure 4:**
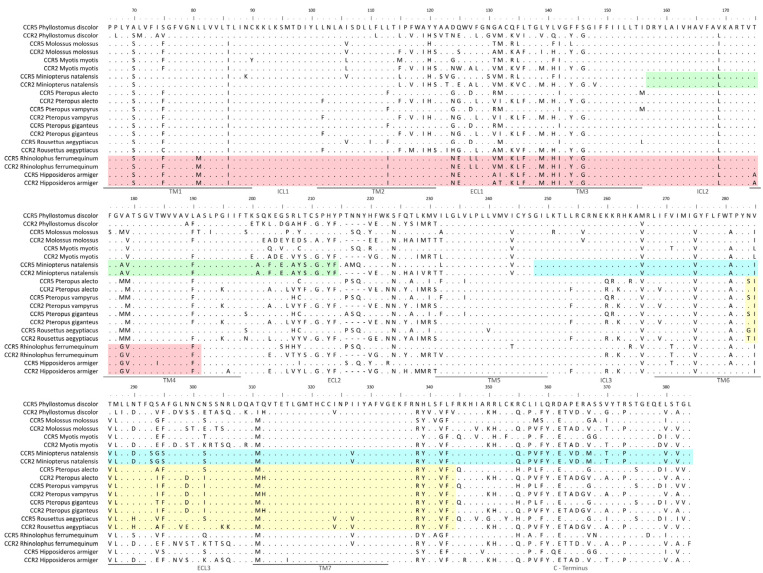
Amino acid alignment of CCR2 and CCR5 from bat species showing different regions where recombination was detected. The CCR5 sequence of *Phyllostomus discolor* was chosen as a reference and identity to this sequence is marked by dots (.). Colored boxes identify the different recombination events detected by RDP [29].

One of the events of gene conversion identified by RDP has affected the CCR2 of all the studied species from the Pteropodidae family (*Pteropus alecto*, *P. vampyrus*, *P. giganteus,* and *Rousettus aegyptiacus*). The region affected by this recombination included residues 284 to 344 of the CCR2 protein in these species, spanning over part of TM6, ECL3, TM7, and part of the C-terminus (Figure 2A). The CCR2 and CCR5 sequences of each Pteropodidae species are not grouped in pairs, but rather follow loosely the expected phylogenetic relations of this family [30], as shown by the phylogenetic tree generated for this alignment region (Figure 3A). This suggests that a single event of recombination has shaped the *CCR2* gene in Pteropodidae before the radiation of this family (~25 mya) [30]. Another event of gene conversion was detected in two species from the Rhinolophoidea superfamily (*Rhinolophus ferrumequinum* and *Hipposideros armiger*). This time, the recombinant gene was identified to be *CCR5*, instead of *CCR2*. The region involved in this recombination was also considerably more extensive, ranging from residues 66 to 191 and spanning over part of TM1, ICL1, TM2, ECL1, TM3, ICL2, and part of TM4 (Figure 2B). In the ML tree generated for this event of gene conversion (Figure 3B), the CCR5 and CCR2 sequences of both *R. ferrumequinum* and *H. armiger* can be seen grouped together within their respective species, contrary to what was observed for the recombination in Pteropodidae. This suggests that concerted evolution is currently acting on CCR2 and CCR5 in Rhinolophoidea, homogenizing the region affected by recombination in CCR5 (Figure 4). Lastly, two events of gene conversion were detected in the CCR5 of *M. natalensis*, explaining the observed proximity between the CCR2 and CCR5 sequences of this species in our initial ML tree (Figure 1). The first event includes residues 157 to 214, which span over ICL2, TM4 and half of the ECL2 regions (Figure 2C). The second event was more extensive, affecting positions 248 to 384 and including part of TM5, ICL3, TM6, ECL3, and TM7 regions, as well as the C-terminus (Figure 2C). *M. natalensis’* CCR2 and CCR5 are identical in the regions affected by the events of recombination (Figure 4), suggesting that concerted evolution is also ongoing there. Overall, our results support that gene conversion was significant in the evolution of CCR2 and CCR5 genes in Chiroptera, having affected large portions of these genes in different clades of bats. The regions of CCR2 and CCR5 affected by gene conversion in this group varied significantly between Chiroptera lineages, including both intracellular and extracellular domains of the two proteins.

In a previous study of recombination between the CCR2 and CCR5 in cats (*Felis catus*) and other lineages of mammals, Vàzquez-Salat et al. [14] have suggested that facilitation of CCR2-CCR5 heterodimerization could provide a selective advantage for the fixation of CCR2-CCR5 chimeric forms. This work only identified recombination to have occurred in structural domains (TMs and ICLs) of both CCR2 and CCR5, which supported their hypothesis since these regions are known to be the most relevant for CCR2-CCR5 heterodimerization [31,32]. However, posterior studies did not find such correlation, and have rather reported that gene conversion events spanned over both structural and functional domains, which is also in line with our results for bats (Figure 2). Furthermore, a more detailed analysis of the *F. catus* CCR2 and CCR5 has shown that gene conversion in this species is also not restricted to TMs and ICLs [17], suggesting that the specificity of recombination between these genes might have been initially overestimated.

Alternatively, host–pathogen interactions have also been proposed as a factor driving the selection of CCR2-CCR5 recombinants [15,16,17]. This hypothesis relies heavily on the existence of pathogens that are able to hijack chemokine receptors and use them to enter the cell. Some examples of such pathogens have indeed been identified, such as HIV-1, dengue virus (DENV), Epstein–Barr virus (EBV), and *Plasmodium vivax* [4,5,6,7,8,9,10,11]. Although DENV and EBV interactions with chemokine receptors remain somewhat elusive, it is well established that both HIV-1 and *Plasmodium vivax* interact directly with chemokine receptors in order to infect the cell [4,5,7,10,11]. Site-directed mutagenesis has shown that HIV infection mainly depends on residues located in extracellular domains of CCR5, such as ECL1-3 and the N-terminus [11,13]. Similarly, *Plasmodium vivax* infection has been shown to rely on the extracellular portion of another chemokine receptor, DARC [7]. The importance of the extracellular domains of chemokine receptors in ligand binding is further supported by evidence of positive selection restricted to these domains [33]. Yet, it must be noted that all events of gene conversion detected in our dataset comprised portions of extracellular domains of either CCR2 or CCR5, although the N-terminus was never part of a recombination. Therefore, it is possible that affected extracellular domains of these chemokine receptors have been selected in bats in order to restrict infection of different pathogens.

Because bats are known to be reservoirs of viruses in nature, it is tempting to speculate that the gene conversion events between CCR2 and CCR5 observed in this group is due to the existence of viruses that have used these proteins as gateways for infection in the past, driving a selective force that has resulted in the fixation of CCR2-CCR5 chimeric forms. However, no virus with these characteristics has yet been identified in bats. Additionally, it has been shown that gene conversion among clustered, closely related genes is not uncommon in nature, suggesting that the fixation of CCR recombinants might be due to random chance [19]. Therefore, whether these recombinant events represent an actual adaptive advantage or not is still dependent on more research. Particularly, by discovering more viruses that use chemokine receptors as a pathway to infection would be of great interest in studying the effects of gene conversion among these proteins and in the adaptive immune system as a whole.

## Data Availability

Not applicable.

## Supplementary Materials

The following are available online at https://www.mdpi.com/article/10.3390/v14020169/s1, Table S1: RDP [29] statistical support (average *p*-value) for each detected recombination.
